# Microcatheter-guided compartment packing of acutely ruptured complex intracerebral aneurysms (ARCIAs): Preliminary experience and technical note

**DOI:** 10.3389/fneur.2022.1020013

**Published:** 2022-11-23

**Authors:** Yi-Bin Zhang, Bing-Sen Xie, Hao-Jie Wang, Sheng-Xuan Huang, Wen-Jian Fan, Mei Zhu, Guo-Rong Chen, Deng-Liang Wang, Pei-Sen Yao, Liang-Hong Yu, Lin-Sun Dai, De-Zhi Kang, Shu-Fa Zheng

**Affiliations:** ^1^Department of Neurosurgery, Neurosurgery Research Institute, The First Affiliated Hospital, Fujian Medical University, Fuzhou, China; ^2^Department of Neurosurgery, National Regional Medical Center, Binhai Campus of the First Affiliated Hospital, Fujian Medical University, Fuzhou, China; ^3^Department of Neurosurgery, Affiliated Sanming First Hospital of Fujian Medical University, Sanming, China; ^4^Department of Neurosurgery, The Affiliated People's Hospital of Fujian University of Traditional Chinese Medicine, Fuzhou, China; ^5^Fujian Provincial Clinical Research Center for Neurological Disease, The First Affiliated Hospital, Fujian Medical University, Fuzhou, China; ^6^Fujian Provincial Institutes of Brain Disorders and Brain Sciences, The First Affiliated Hospital, Fujian Medical University, Fuzhou, China; ^7^Clinical Research and Translation Center, The First Affiliated Hospital, Fujian Medical University, Fuzhou, China

**Keywords:** endovascular embolization, intracerebral aneurysm, subarachnoid hemorrhage, microcatheter, treatment, prognosis

## Abstract

**Objective:**

We present our initial experience using the microcatheter-guided compartment packing (MCP) technique for endovascular embolization of acutely ruptured complex intracerebral aneurysms (ARCIAs) and evaluate the safety, feasibility, and efficiency of this technique.

**Methods:**

This retrospective, single-center study included 28 patients who underwent coil embolization using the MCP technique for ARCIAs at our institution between January 2021 and January 2022. The MCP technique was the placement of microcatheters in different compartments within the aneurysm to deploy the coils simultaneously or sequentially. Patient demographics, aneurysm characteristics, procedural parameters, grade of occlusion, complications, and clinical results were analyzed. The clinical outcomes were evaluated with modified Rankin Scale (mRS) scores.

**Results:**

Of the 28 patients successfully treated with the MCP technique, 24 (85.7%) aneurysms were considered as complete occlusions (Raymond I) based on the immediate postembolization angiogram results. Complications occurred in 2/28 treatments, including guidewire perforation with subarachnoid hemorrhage and cerebral vasospasm-related cerebral infarction. An angiography follow-up demonstrated complete occlusion in 25/28 aneurysms. Twenty-six (92.9%) patients had favorable 90-day outcomes (mRS 0-2) after the endovascular coil embolization.

**Conclusion:**

The MCP technique is simple, safe, and effective, achieving good packing density and initial occlusion rate when used to treat ARCIAs.

## Introduction

Complex intracerebral aneurysms (CIAs) are irregular, large or giant, wide-necked or combined with arterial branches, perforating vessels, atheromatous vessels, and partially thrombosed (1–3). The acutely ruptured complex intracerebral aneurysms (ARCIAs) result in devasting consequences, and the treatment of patients with ARCIAs has been cited as the most vexing scientific issue confronting neurosurgeons and neuroradiologists. Endovascular treatment of CIAs has become an effective alternative to standard surgical clipping (4–7). Despite the advances in endovascular technologies, the treatment of ARCIAs is considered more challenging due to the difficulties in positioning the microcatheter, the coil matrix's lack of support, the increased risk of parent vessel compromise, and the inability to pack the aneurysm densely compared with classic aneurysms with narrow necks (1, 3, 5).

Emerging data suggest that coil embolization may result in 50% to 60% recanalization rates in CIAs (8, 9). In endovascular coil embolization of ARCIAs, dense coil packing of the aneurysm sac is crucial in preventing recurrence (10, 11). To achieve the densest possible coil packing, it is essential to correctly select the appropriate embolization strategy. A growing body of literature reports using multi-microcatheter, stent coiling, microcatheter shaping, and balloon assistance for intracerebral aneurysms (IAs) embolization with good clinical follow-up results (12–15). Recent retrospective studies have reported that the dual microcatheter technique is a safe and effective treatment for acutely ruptured aneurysms due to low treatment-related complications and mortality (16–18). Although these studies are informative, most of these studies focus on describing the results of the studies and do not specifically address the selection of microcatheters for IAs embolization and the rationale for the selections. In addition, prior literature demonstrated the use of microcatheters to temporarily alter the configuration of the aneurysm neck and bifurcation branches (also known as microcatheter protection techniques) to assist in aneurysm embolization, rather than direct embolization of the aneurysm using microcatheter coil delivery (15, 19).

Herein, we presented our initial experience with a microcatheter-guided compartment packing (MCP) technique for ARCIAs. The MCP technique was that the distal tips of the microcatheter were cast into different shapes based on the aneurysm type, shape, and dome orientation, and the tips of microcatheters were placed in a different compartment within the aneurysm to deploy the coils simultaneously or sequentially. The intraoperative microcatheter was adjusted according to the embolization strategy, and the microcatheters might be adjusted according to the embolization area of the expected aneurysm, even with the single microcatheter technique. Additionally, more 3-dimensional (3-D) coils were used intraoperatively to be woven into a basket based on preset microcatheter varying with the size and shape of the aneurysm since coil selection is an essential factor in successful embolization and postoperative recurrence (16). The MCP technique enables neurointerventionalists to effectively lock the coil to form a stable frame, reduce the likelihood of coil protrusion, and achieve a high-volume embolization ratio for ARCIAs.

## Materials and methods

### Study population

The Institutional Review Board approved this retrospective analysis of the First Affiliated Hospital of Fujian Medical University. The medical records of the patients with acutely ruptured complex intracerebral aneurysms (ARCIAs) were treated with the MCP technique at our institution between January 2021 and January 2022. Patients were eligible when (i) diagnosis of the ruptured intracerebral aneurysm (IA) by computed tomography angiography (CTA) or/and digital subtraction angiography (DSA). CTA or/and DSA were conducted before the endovascular procedure to determine the size and location of IA. (ii) Patients who met the diagnostic criteria of CIAs. According to the published literature, CIAs were defined as irregular, large or giant, wide neck or combined arterial branches, perforating vessels, atherosclerotic vessels, and partial thrombosis (1, 3, 20). Wide-necked aneurysms were those with a diameter greater than 4 mm or a dome-to-neck ratio less than 2 (20). (iii) ARCIAs were treated with the MCP technique upon admission or within 72 h of hospital admission. Exclusion criteria were as follows: (i) patients with multiple aneurysms; (ii) patients who died during follow-up; (iii) those who could not undergo another DSA or magnetic resonance angiography (MRA) because of other diseases; (iv) those who refused to undergo a DSA or MRA review; (v) patients with pre-treatment modified Rankin Scale (mRS) score >2. In this study, Hunt-Hess grade 4-5 was defined as a severe clinical condition and mFisher grade 3-4 was considered severe aneurysmal subarachnoid hemorrhage (aSAH) (21). Neurointerventionalists and neurosurgeons immediately accessed all patients after initial CTA or/and DSA, and the mode of therapy was chosen consensually.

**Figure 1:**
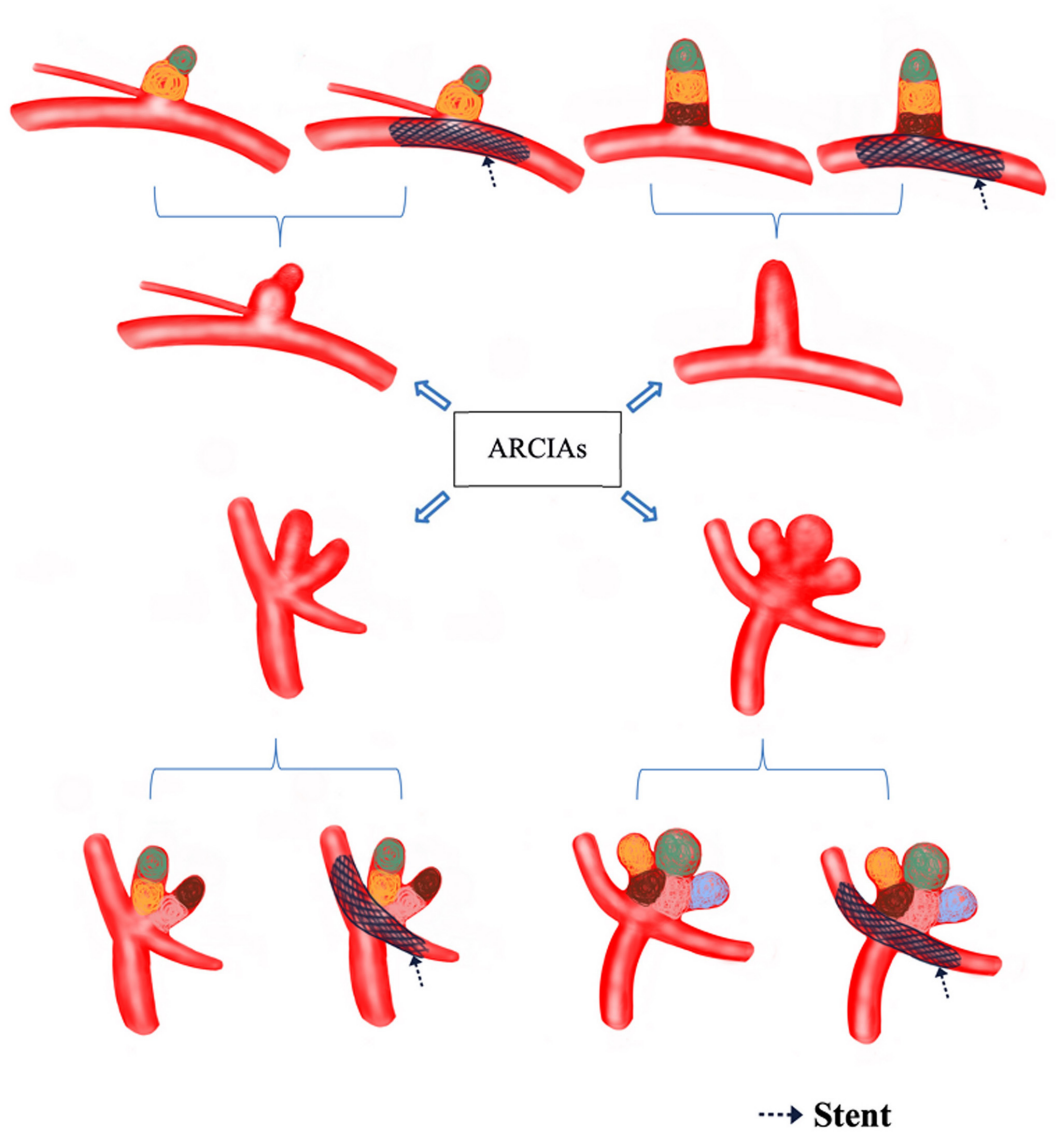
Decision flow chart for treating ARCIAs. Black arrows indicate the stents (which can also be balloons) that assist in compartment packing. The different colored circles represent compartment packing in different compartments. ARCIAs, acutely ruptured complex intracranial aneurysms.

All patients were treated as a priority with endovascular coil embolization. Figure 1 illustrates the decision flow chart for treating ARCIAs. Immediate radiological outcomes were analyzed. Procedure-associated complications were defined as any outcomes related to the procedure, such as hemorrhage, infarction, or dissection, which was life-threatening or resulted in permanent morbidity or mortality or any unexpected event that caused persistent or significant disability (22). Neurological outcome was assessed using the mRS score before the procedure and at discharge. The mRS score was categorized into favorable (mRS 0-2) or unfavorable (mRS 3-6) (21).

### Angiographic results

Angiography was graded according to the Raymond scale (23). The degree of occlusion following embolization was classified into three classes: Raymond I (complete occlusion, including the neck), Raymond II (residual neck), and Raymond III (residual aneurysm sac) (23). Follow-up angiographic examinations were performed at 6 months post-embolization with DSA or MRA.

### Technical note

The interventional procedures were performed under general anesthesia. After a successful puncture through the right femoral arteries using the Seldinger technique, a 6F or 8F arterial catheter sheath was placed on the right side, and the patients received intravenous systemic heparinization. A 6 or 8-Fr guiding catheter was connected to a Y-valve with one or two double-head Y-valves in the petrous internal carotid artery for anterior circulation aneurysms or vertebral artery at the C_2_ spine level for posterior circulation aneurysms. First, cerebral angiography was performed to evaluate interventional treatment pathways, arteriosclerosis, stenosis, and collateral circulation. Aneurysm height, transverse diameter, neck sizes, the number of substances or lobules, and the positional relationship between the parent artery and the aneurysm, as well as proximal and distal parent artery diameter, were obtained upon review of procedural images, which included DSA and 3-dimensional (3D) rotational angiography reconstructions. These were the basis for implementing MCP technology. Aneurysm dimensions were measured by 3D images derived from rotational angiography. The optimal working angle was determined by analyzing the 3D reconstruction results and the embolization strategies, including the shape and angle of the microcatheter and the need for stent assistance. Second, according to the shape and size of the aneurysm, the morphology of the true aneurysm and the sac, the relationship between the aneurysm and the parent artery, and the order of compartment packing were determined. When necessary, precise shaping of the microcatheter tip with steam was performed to improve navigability and stability. The principle of shaping the tip of the microcatheter was that it could enter the first partition smoothly and then select the second microcatheter for shaping to enter the second partition. Multiple microcatheters may be required if ARCIAs have multiple compartments, which can not be satisfied by a single or dual microcatheter. In addition, individual microcatheters should be able to enter the second or even the third partition smoothly. The microcatheters would occupy different parts of the aneurysm. Care must be taken when positioning the second microcatheter, as it can cause the first microcatheter to migrate forward. Under the 3D Roadmap, the microcatheters were slowly advanced into the daughter sac of the target partition *via* a 0.014-inch micro guidewire to perform the MCP.

Under “roadmap” conditions, coils were deployed into the aneurysm. The first 3D coil that matched the target sac size was woven into a basket on a preset microcatheter varying the size and shape of the aneurysm, and the size of the coils was successively decreased to perform embolization of the target partition until it was compactly embolized. Coil-assisted embolization can be used for aneurysms with multiple daughter sacs and wide necks. The rims in the second partition must climb several times within the first partition to increase the stability of the rims in the second partition. If stent-assisted embolization of IA is required, the Jailing, semi-Jailing, or Mesh technique can be used depending on the situation (24–27). If a stent-assisted embolization was necessary for patients with ARCIAs, tirofiban was administered intravenously before the stent was released. After the interventional operation, continuous drainage of the lumbar cistern or lumbar puncture was performed to release bloody cerebrospinal fluid (CSF) according to the amount of bleeding on brain CT and the Hunt-Hess grade. All surgical procedures and perioperative management followed the institutional protocols which were based on current guidelines (28).

**Figure 2:**
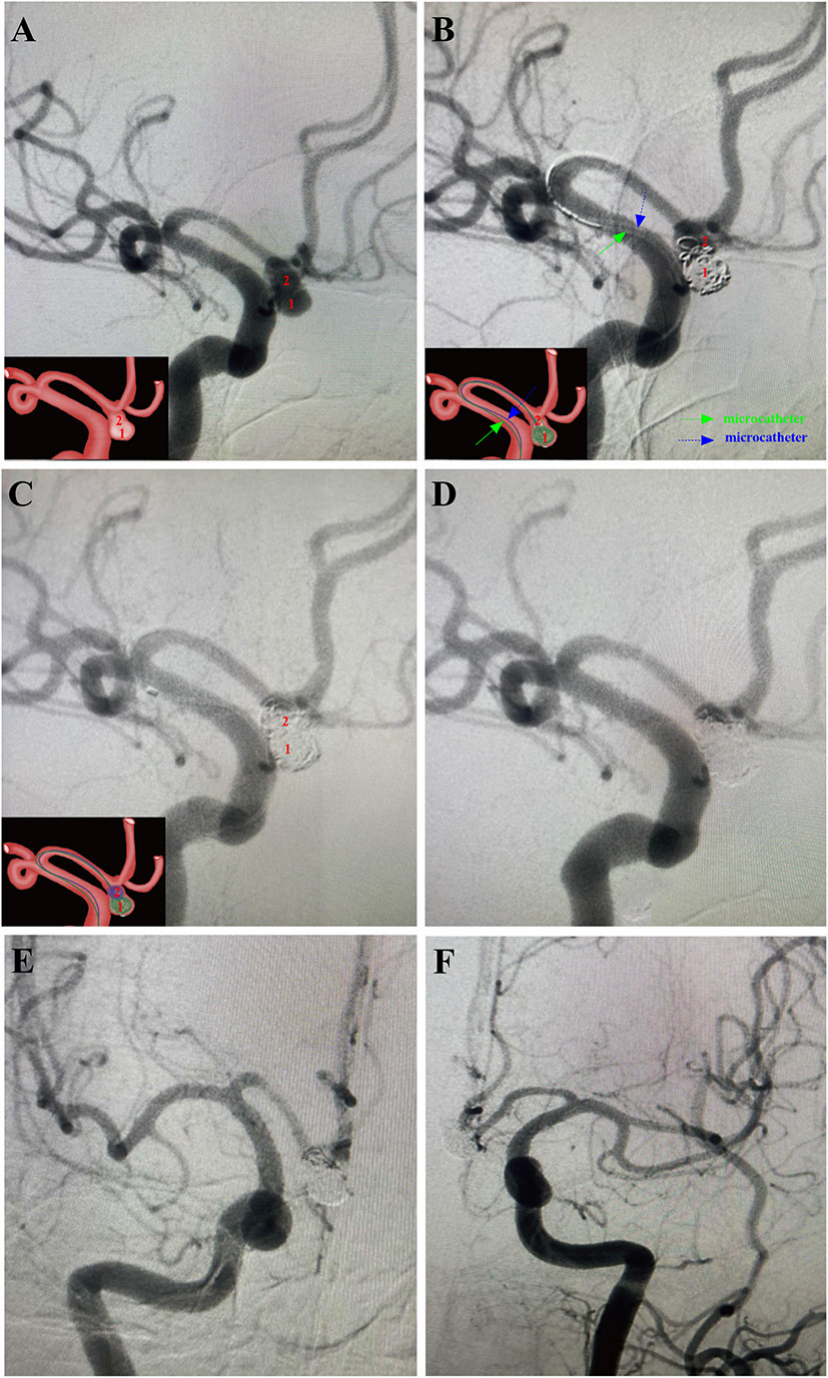
A 54-year-old male patient with a sudden headache for 7 h was admitted into our department with the diagnosis of subarachnoid hemorrhage (Hunt-Hess grade II) arising from ruptured irregular AComA. Then, this aneurysm was successfully treated using the MCP technique in the acute stage. Arabic numerals indicate the ascus of an IA. Right ICA DSA (working angle) demonstrated a gourd-like irregular aneurysm with a daughter sac at the AComA. Double microcatheters were introduced in the IA sac. Embolization was performed with a microcatheter (green arrow) navigating into the lower daughter sac and coils packing. Embolization with coils of appropriate size alternatively through the tips of the two microcatheters until embolism was satisfactory. After packing the lower sac, the upper daughter sac was coiled *via* the other microcatheter (blue arrow). **(D–F)** Post-embolization digital subtraction angiograms exhibited occlusion at Raymond grade II. **(D)** The working angle view of postoperative embolization. **(E)** The anteroposterior view of the right ICA after postoperative embolization. **(F)** The anteroposterior view of the left ICA after postoperative embolization. The inserts in the lower right corner illustrated an aschematic diagram of the MCP process of the IA **(A–C)**.

**Figure 3:**
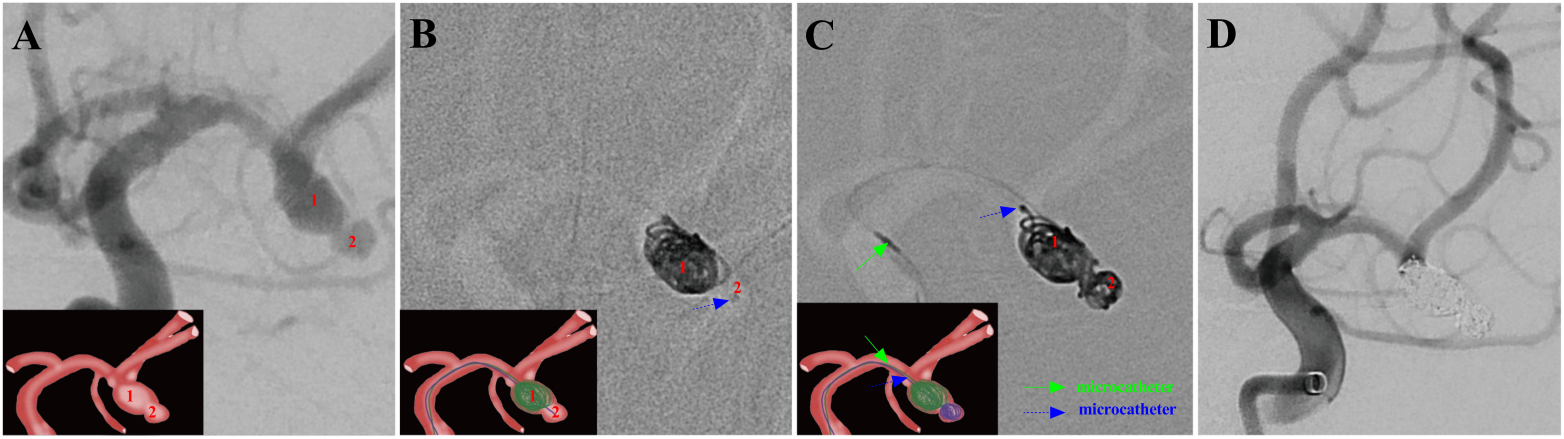
A ruptured right MCA aneurysm (Hunt-Hess grade II) in a 46-year-old female was treated using the MCP technique. Arabic numerals indicate the ascus of an IA. **(A)** Right ICA DSA (working angle) displayed a gourd-like irregular aneurysm with a daughter sac at the MCA. **(B)** Dual microcatheters were introduced in the IA sac. Embolization was performed with a microcatheter (blue arrow) navigating into the upper daughter sac and coils packing (image of digital non-subtraction angiography). **(C)** After packing the upper sac, the lower daughter sac was coiled *via* the other microcatheter (green arrow). **(D)** The working angle view of postoperative embolization showed complete occlusion of MCA aneurysm (Raymond grade I).

**Figure 4:**
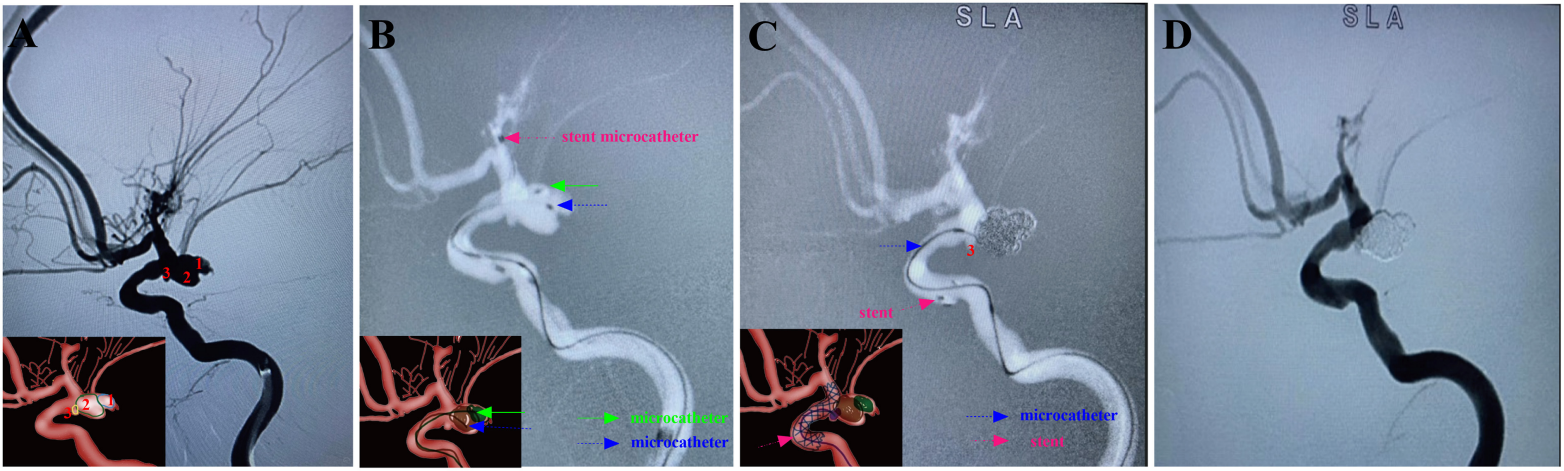
A ruptured right PcomA aneurysm (Hunt-Hess grade IV) in a 66-year-old female was treated using the stent-assisted MCP technique. In addition, the patient had coexisting Moyamoya disease. Arabic numerals indicate the ascus of an IA. **(A)** DSA (working angle) showed a cauliflower-shaped irregular aneurysm of PcomA with three daughter sacs. **(B)** The stent-microcatheter (pink arrow) was placed at the beginning of the M1 segment of the MCA. Double-coil microcatheters (blue and green arrows) were introduced in different sacs of the IA at appropriate surgical angles. **(C)** We used the Jailing technique to coil the aneurysm with the assistance of an Enterprise 2 stent (Codman Neurovascular, Raynham, Massachusetts, USA). Embolization was first performed with a microcatheter (green arrow) navigating into the upper daughter sac and coils packing (image of digital non-subtraction angiography). The coils were passed through the other microcatheter (blue arrow) to embolize the middle sacs, and then the microcatheter was withdrawn into the lower sac for coil embolization. **(D)** Post-embolization digital subtraction angiograms at the working angle exhibited complete occlusion at Raymond grade I. The inserts in the lower right corner illustrated an aschematic diagram of the MCP process of the IA **(A–C)**.

**Figure 5:**
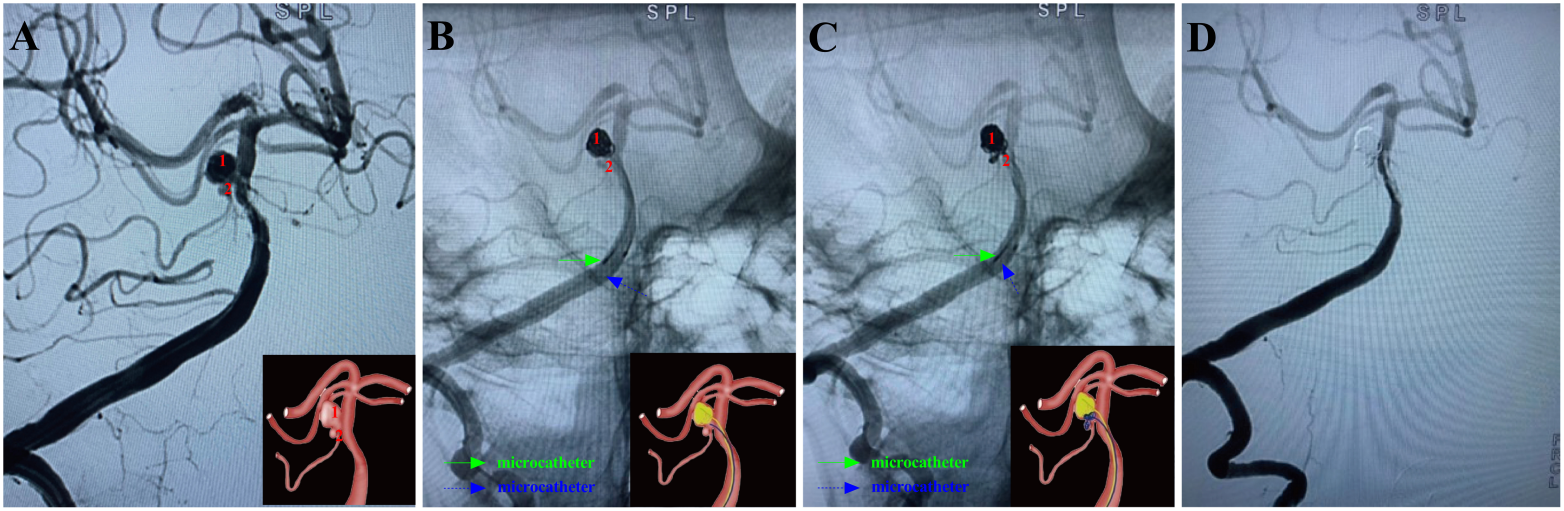
A ruptured right anterior inferior cerebellar artery aneurysm (Hunt-Hess grade III) in a 59-year-old female was treated using the MCP technique. Arabic numerals indicate the ascus of an IA. **(A)** Right vertebral artery digital subtraction angiography (working angle) showed an irregular aneurysm with a daughter sac at the bifurcation of the basilar artery and right superior cerebellar artery. **(B)** Double-coil microcatheters (blue and green arrows) were introduced in different sacs of the IA. Embolization was first performed with a microcatheter (green arrow) navigating into the upper daughter sac and coils packing (image of digital non-subtraction angiography). The other microcatheter completed the remaining part of the bottom of the aneurysm sac (blue arrow). **(C)** The other microcatheter completed the remaining part of the bottom of the aneurysm sac (blue arrow). After packing the upper sac, the microcatheter (blue arrow) enters the lower sac. Subsequently, the lower daughter sac was coiled (blue arrow). **(D)** Post-embolization digital subtraction angiograms at the working angle exhibited complete occlusion at Raymond grade I. The inserts in the lower right corner illustrated an aschematic diagram of the MCP process of the IA **(A–C)**.

Here, examples of clinical application of the MCP technique for ARCIAs embolization were illustrated in Figures 2–5.

## Results

Twenty-eight patients presenting with subarachnoid hemorrhage (SAH) secondary to ruptured complex intracerebral aneurysms (RCIAs) were enrolled in treatment with the MCP technique based on the inclusion criteria between January 2021 and January 2022. Patient demographics and aneurysm characteristics are summarized in Table 1. This study included 28 patients (20 women and 8 men) aged from 29 to 79 years, with a mean age of 55.96. Five (17.9%) patients were in severe clinical condition (Hunt-Hess grade 4–5), and five (17.9%) patients had severe aSAH (mFisher grade 3–4) on admission. The aneurysms had sizes ranging from 2.80 to 19.87 mm (mean 7.53 mm), necks of 4.10 to 10.50 mm (mean 5.96 mm), and dome-to-neck ratios of 0.51 to 4.01 (mean 1.29). Twenty-one (75.0%) aneurysms presented irregular shapes. Sixteen (64.3%) aneurysms were located in the anterior circulation, and the other 10 (35.7%) were in the posterior circulation. Pre-treatment mRS score was 0.

**Table 1:** Patient demographics and aneurysm characteristics.

**Characteristic**	**Value**
	
Number of patients	28
Age (years)	29–79 (mean, 55.96)
**Sex (** ***N*** **, %)**
Male	8 (28.6)
Female	20 (71.4)
**Hunt-Hess grade (** ***N*** **, %)**
1–3	23 (82.1)
4–5	5 (17.9)
**mFisher grade (** ***N*** **, %)**
1–2	23 (82.1)
3–4	5 (17.9)
**Aneurysm location (** ***N*** **, %)**
AComA /ACA	4 (14.3)
MCA	2 (7.1)
PcomA/Anterior choroidal artery	6 (21.4)
ICA bifurcation	6 (21.4)
Basilar bifurcation	4 (14.3)
VA/AICA/PICA	6 (21.4)
**Aneurysm characteristics**
Aneurysm size (mm)	2.80–19.87 (mean, 7.53)
Neck size (mm)	4.10–10.50 (mean, 5.96)
Dome-to-neck ratio	0.51–4.01 (mean, 1.29)
Irregularity (N,%)	21 (75.0)
**mRS at 90-day (** ***N*** **, %)**
0–2	26 (92.9)
3–6	2 (7.1)

ACA, anterior cerebral artery; ACoA, anterior communicating artery; MCA, middle cerebral artery; PcomA, posterior communicating artery; ICA, internal carotid artery; VA, vertebral artery; AICA, anterior inferior cerebellar artery aneurysm; PICA: posterior inferior cerebellar artery; mRS: modified Rankin Scale.

Endovascular embolization of ARCIAs using the MCP technique showed technical success in all patients. Seventeen (60.7%) aneurysms were treated with the double-microcatheter MCP technique, and 11 (39.1%) were treated with the single-microcatheter MCP technique (Table 2). Thirteen (46.4%) aneurysms were treated with coil only, and 15 (53.6%) aneurysms received stent-assisted coiling. Of 28 patients treated with the MCP technique, 24 (85.7%) aneurysms were considered complete occlusion (Raymond I) based on the immediate postembolization angiogram results (Table 2). In contrast, four (14.3%) aneurysms were considered Raymond II. Figures 2–5 illustrate anterior communicating artery (AComA), middle cerebral artery (MCA), posterior communicating artery (PcomA), and anterior inferior cerebellar artery (AICA) aneurysms embolization process.

**Table 2:** Immediate post procedural angiographic results and clinical complications.

**Characteristic**	**Value**
	
**Microcatheter technique (** ***N*** **, %)**
Single-microcatheter	11 (39.3)
Double-microcatheter	17 (60.7)
Multiple-microcatheter	0 (0.0)
**Stent-assisted coiling (** ***N*** **,%)**	15 (53.6)
**Postembolization angiography (** ***N*** **, %)**
Raymond I	24 (85.7)
Raymond II	4 (14.3)
Raymond III	0 (0.0)
**Adverse events (** ***N*** **, %)**
Hemorrhagic	1 (3.57)
Ischemic	1 (3.57)

Complications occurred in 2/28 treatments, including guidewire perforation with SAH (directly related to the procedure) and cerebral vasospasm-related cerebral infarction. Aneurysm perforation occurred due to trauma caused by the pushing guide wire of a small daughter sac located above the treated aneurysm. As a result, the patient developed a small SAH. Postoperatively, the patient presented with headache symptoms, which almost wholly subsided after treatment, and the mRS score at discharge was 0. The DSA or MRA follow-up at 6 months post-embolization was available for all 28 survivors, demonstrating complete occlusion in 25/28 (89.3%) aneurysms, and the recanalization rate was 3.57% (Table 3). Twenty-six (92.9%) patients had a favorable 90-day outcome (mRS 0–2) after the endovascular coil embolization.

**Table 3:** Follow-up angiographic results.

**Follow-up**	**Postembolization**			**Total**
**angiography**	**angiography**		
	**Raymond I**	**Raymond II**	**Raymond III**	
Raymond I	24	1	0	25
Raymond II	0	2	0	2
Raymond III	0	1	0	1
Total	24	4	0	28
Recanalization	0/24	$\frac{1}{4}$ (25%)	0	1/28 (3.57%)

## Discussion

**Figure 6:**
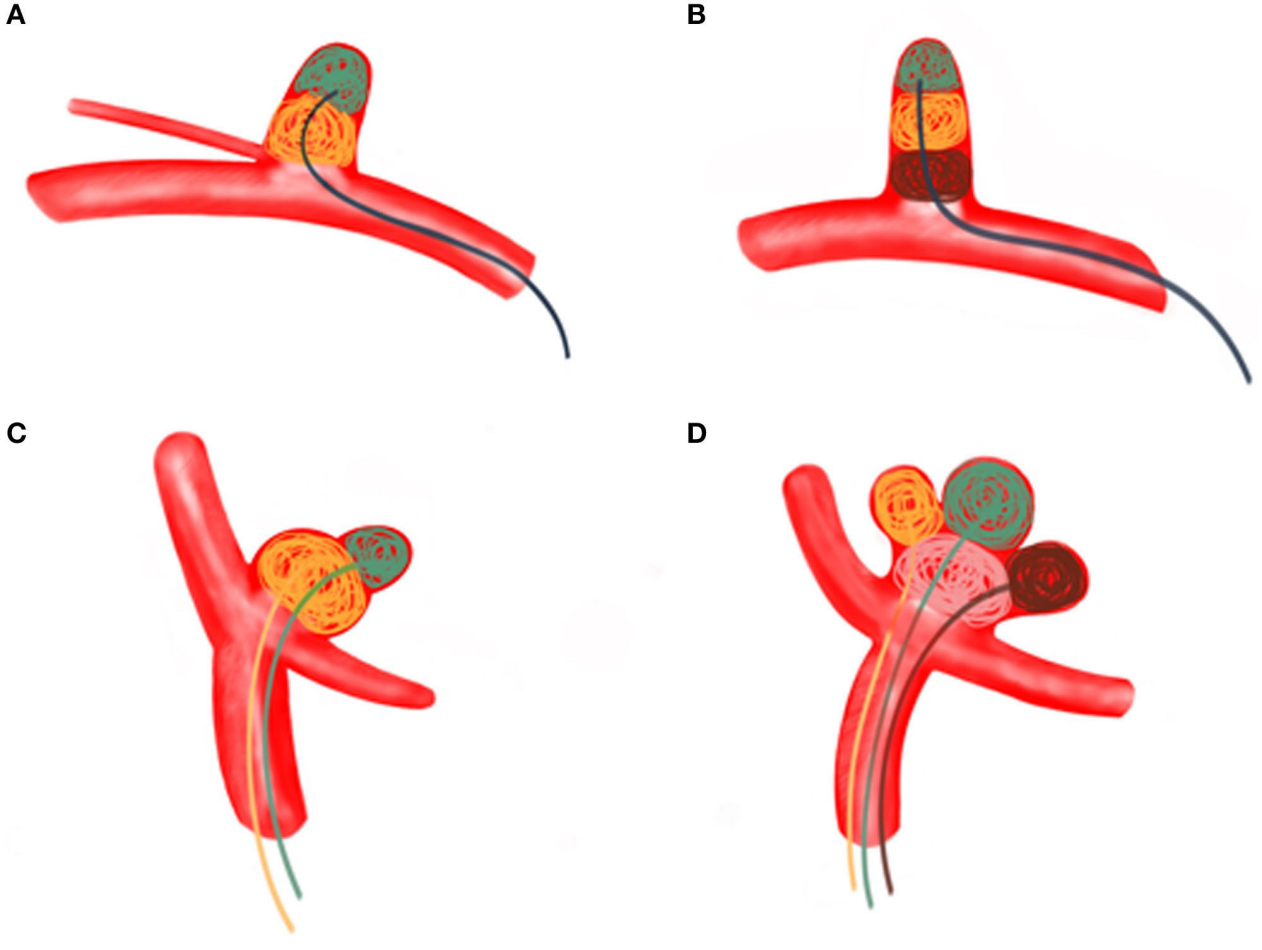
**(A)** A regular wide-necked aneurysm was treated using the MCP technique with a single microcatheter. Aneurysm embolization proceeds from the distal part of the aneurysm to the proximal part. **(B)** The MCP technique of a single microcatheter was used for long, regular arteries involving branch vessels. Aneurysm embolization proceeds from the distal part of the aneurysm toward the proximal part. **(C)** Dual microcatheter MCP technique was used to treat bifurcated irregular polysaccular aneurysms. The small ruptured sac is embolized first, followed by the embolization of the larger sac (the aneurysm). **(D)** Irregular polysaccular aneurysm was treated with multi-microcatheter MCP technique. Aneurysm embolization is performed from the individual sub-sacs of the aneurysm.

ARCIAs have always been a tremendous challenge for surgical clipping and endovascular coiling due to their complex vascular anatomy. In clinical practice, we introduced an MCP technique for ARCIAs and applied it to aneurysm embolization. Here, we listed four clinical applications of the MCP technique for ARCIAs embolization (Figures 2–5). The principal findings obtained in the present study are as follows. Of 28 patients successfully treated with the MCP technique, 24 (85.7%) aneurysms were considered as complete occlusions, and an angiography follow-up at 6 months post-embolization demonstrated complete occlusion in 25/28 aneurysms. The rate of recanalization at 6 months post-embolization was 3.57%. Moreover, we proposed a hypothetical model diagram in Figure 6 to embolize an aneurysm using the MCP technique, where the microcatheters were placed in a different compartment within the aneurysm to deploy the coils simultaneously or sequentially. Our report presents initial clinical experiences with the MCP technique, which provides a safe, reliable, and practical approach to the endovascular embolization of ARCIAs.

Several pieces of literature have demonstrated the use of microcatheters to temporarily alter the configuration of the aneurysm neck and bifurcation branches (also known as microcatheter protection techniques) to assist in aneurysm embolization (15, 16, 19). The present study differs from the previous literature in several ways. First, the MCP technique used in our case series was used to deliver coils. Second, our case series illustrated that the intraoperative microcatheter is adjusted according to the embolization strategy, and the microcatheter can be adjusted according to the embolization area of the expected aneurysm, even with the single microcatheter technique. Third, more 3-dimensional (3-D) coils were used intraoperatively to be woven into a basket based on a preset microcatheter varying with the size and shape of the aneurysm in our case series. Finally, coil-assisted embolization can be used for aneurysms with multiple daughter sacs and wide necks. The rims in the second partition must climb several times within the first partition to increase the stability of the rims in the second partition.

Compared with surgical clipping, endovascular treatment has become a preferred method in most cases for treating unruptured and ruptured IAs—over 50% of IAs are treated *via* endovascular coiling (7, 29, 30). Difficulties in traversing the microcatheter through tortuous vasculature, guiding the microcatheter tip to the 1/2 or 2/3 position within the aneurysm, and maintaining it in a stable position account for many coil embolization procedures fail. These causes include incomplete aneurysm occlusion (16, 31, 32), aneurysm recanalization (31), coil misalignment (33), and intraoperative rupture (34). These failures can be attributed to unfavorable vessel tortuosity and CIAs geometry. CIAs of varying shapes and sizes, and the stability of the intra-aneurysm embolization coil after intervention remain challenges for endovascular embolization techniques (7). Based on the MCP technique, the neurointerventionalist may advance the coil filling at the working projection without hesitation and may not worry about moving the microcatheter into the parent vessel or filling the coil into the parent vessel.

The MCP technique has several unique strengths. First, it exploits the superior tracking capabilities of the microcatheter, allowing flexible and maneuverable embolization of aneurysms in tortuous vasculature or smaller distal arteries. This technique can avoid remodeling and repositioning the microcatheter later and reduce surgical time and postoperative complications. Second, the MCP technique facilitates intraoperative selection of coil size. In the compartments of the aneurysm, the coils are gradually and orderly piled up, stabilizing the coils, and making them less likely to come out of the vessel. On the other hand, the microcatheter is simultaneously placed in the aneurysm sac, keeping the coils in place, and intertwined to reduce the chance of protruding. Third, the MCP technique facilitates dense embolization. It allows placing many coils in the aneurysm sac, creating a stable build before separating them. During the procedure, the distal tips of the microcatheter are steam-shaped in different shapes according to the shape of IA, and the tips of the microcatheters are implanted in different parts of IAs. ARCIAs are then densely embolized by using the microcatheters simultaneously or alternately. It should be noted that when stent-assisted embolization is used, anti-platelet aggregation drugs such as tirofiban must be used. At this time, forming an intratumoral thrombus in the aneurysm is complex, and ARCIAs can achieve the purpose of dense embolization of the aneurysm with the MCP technique. On the other hand, if stent-assisted embolization is necessary, using the MCP technique can effectively reduce coils escaping from the aneurysm cavity, increase coil packing density, and reduce aneurysm recurrence. In a literature review of stent-assisted coil embolization for ARCIAs, 63% of aneurysms exhibited complete occlusion (35). A published series with a large population using stent-assisted coil embolization for ARCIAs showed immediate occlusion in 87.5% of patients (36–39). From a multitude of perspectives, when coils are loosely packed on ruptured aneurysms, they appear to increase the risk of recurrent SAH, re-ruptured, and postoperative recurrence (15, 16). In the present study, satisfactory aneurysm occlusion was achieved in 89.3% of cases at 6 months postembolization, and the recanalization rate of 3.57% was low. The MCP technique can effectively increase the coil packing density, reduce perioperative re-rupture or rebleeding, and minimize the risk of postoperative recurrence.

The MCP treatment for ARCIAs is a double-edged sword, raising three serious concerns. First, placing two or more microcatheters into the aneurysm may increase the risk of rupture. In this study, a guidewire-induced aneurysm perforation occurred, resulting in SAH. The previous literature reported that the incidence of rebleeding caused by two or more microcatheters was 3% (14, 15), which is similar to the 2.0% to 7.3% reported incidence for all aneurysms regardless of size or presence of subarachnoid hemorrhage (14, 31). Second, coiling ARCIAs without a stent or balloon may increase the risk of coil protrusion and parent artery occlusion (36–40). Although no similar complication occurred in our cases, it still requires concern. Third, coiling ARCIAs without stent or balloon assistance may reduce packing density and increase recurrence rates (36, 38–41). In our case, complete occlusion (Raymond I) was achieved in 89.3% of cases at 6 months postembolization, and we look forward to long-term follow-up results. Taken together, neurointerventionalists need to carefully balance the risk of aneurysm recurrence with the inherent risk of stent or balloon remodeling. Nevertheless, it is undeniable that stent-assisted coil embolization has been increasing for ARCIAs (25, 42).

Notably, not all ARCIAs can be treated endovascularly. Up to 25% of surgical neurointerventions fail in attempted endovascular treatment of intracranial aneurysms (7). Some ARCIAs require revascularization techniques, including extracranial-to-intracranial carotid artery bypass with aneurysm clipping or distal or proximal arterial occlusion, intracranial-to-intracranial bypass, and excision of the diseased segment with direct vessel revascularization (*in situ* bypass) (1, 43). Future studies are needed to explore whether the MCP technique can guide the choice of surgical modalities for the revascularization of such CIAs. Moreover, we did not obtain a long-term follow-up result in our patients even though we obtained a good packing density and stable short-term outcome. Further follow-up and application in more patients will be required. Third, since the sample size in the present study was small, larger-scale clinical trials are needed to verify the generalizability of the present findings. Fourth, for treating giant aneurysms, the use of MCP technology requires the use of bilateral femoral artery sheath and multi-catheter system technology, which may increase surgical complexity, heighten the risk of intraoperative complications, and increase operative times.

## Conclusion

The MCP technique is simple, safe, and effective, achieving good packing density and initial occlusion rate when used for the treatment of ARCIAs, and may serve as an option for treating ARCIAs. Further studies are necessary to evaluate its long-term effectiveness.

## Data availability statement

The original contributions presented in the study are included in the article/supplementary material, further inquiries can be directed to the corresponding authors.

## Ethics statement

The studies involving human participants were reviewed and approved by the Ethics Committee of the First Affiliated Hospital of Fujian Medical University. The Ethics Committee waived the requirement of written informed consent for participation.

## Author contributions

Y-BZ, D-ZK, and S-FZ designed the study. Y-BZ, B-SX, H-JW, and S-XH collected, analyzed data, and drafted the manuscript. W-JF, MZ, G-RC, P-SY, L-SD, and L-HY helped with the figures and result in interpretation. D-LW, L-SD, L-HY, D-ZK, and S-FZ reviewed and revised the manuscript. L-SD, D-ZK, and S-FZ were identified as the guarantor of the article, taking responsibility for the integrity of the work as a whole. All authors contributed to the article and approved the submitted version.

## Funding

The study was supported by the National Natural Science Foundation of China (Nos. 81870930 and 82171327) and Qihang Fund Project of Fujian Medical University (2019QH1262).

## Conflict of interest

The authors declare that the research was conducted in the absence of any commercial or financial relationships that could be construed as a potential conflict of interest.

## Publisher's note

All claims expressed in this article are solely those of the authors and do not necessarily represent those of their affiliated organizations, or those of the publisher, the editors and the reviewers. Any product that may be evaluated in this article, or claim that may be made by its manufacturer, is not guaranteed or endorsed by the publisher.
